# Magnitude of income-related disparities in adverse perinatal outcomes

**DOI:** 10.1186/1471-2393-14-96

**Published:** 2014-03-04

**Authors:** Ketan Shankardass, Patricia O’Campo, Linda Dodds, John Fahey, KS Joseph, Julia Morinis, Victoria M Allen

**Affiliations:** 1 Department of Psychology, Wilfrid Laurier University, 75 University Avenue West, Waterloo, Ontario, Canada; 2 Centre for Research on Inner City Health, St. Michael’s Hospital and Dalla Lana School of Public Health, University of Toronto, Toronto, Ontario, Canada; 3 Department of Obstetrics and Gynaecology, Dalhousie University, Halifax, Nova Scotia, Canada; 4 Reproductive Care Program of Nova Scotia, Halifax, Nova Scotia, Canada; 5 Department of Obstetrics & Gynaecology, School of Population and Public Health, University of British Columbia and the Children’s and Women’s Hospital of British Columbia, Vancouver, British Columbia, Canada; 6 Department of Paediatric Medicine, Hospital for Sick Children, Toronto, Ontario, Canada

**Keywords:** Perinatal, Socioeconomic position, Health inequalities, Neighborhood, Income

## Abstract

**Background:**

To assess and compare multiple measurements of socioeconomic position (SEP) in order to determine the relationship with adverse perinatal outcomes across various contexts.

**Methods:**

A birth registry, the Nova Scotia Atlee Perinatal Database, was confidentially linked to income tax and related information for the year in which delivery occurred. Multiple logistic regression was used to examine odds ratios between multiple indicators of SEP and multiple adverse perinatal outcomes in 117734 singleton births between 1988 and 2003. Models for after tax family income were also adjusted for neighborhood deprivation to gauge the relative magnitude of effects related to SEP at both levels. Effects of SEP were stratified by single- versus multiple-parent family composition, and by urban versus rural location of residence.

**Results:**

The risk of small for gestational age and spontaneous preterm birth was higher across all the indicators of lower SEP, while risk for large for gestational age was lower across indicators of lower SEP. Higher risk of postneonatal death was demonstrated for several measures of lower SEP. Higher material deprivation in the neighborhood of residence was associated with increased risk for perinatal death, small for gestational age birth, and iatrogenic and spontaneous preterm birth. Family composition and urbanicity were shown to modify the association between income and some perinatal outcomes.

**Conclusions:**

This study highlights the importance of understanding the definitions of SEP and the mechanisms that lead to the association between income and poor perinatal outcomes, and broadening the types of SEP measures used in some cases.

## Background

In Nova Scotia, Canada, despite all families having access to essential health services through a publicly funded insurance program, lower-income mothers have worse perinatal outcomes than mothers with higher income [1]. Socioeconomic position (SEP) is a multi-dimensional characteristic with an indirect, complex relationship to perinatal health [1-5], and past studies indicate that multiple indicators should be considered when measuring inequalities [6,7].

Effects associated with SEP may reflect unique and inter-related mechanisms at multiple levels. This includes maternal and family characteristics associated with low income that mediate effects on adverse perinatal outcomes (e.g., whether or not a mother uses tobacco products during pregnancy), as well as macrosocial factors (i.e., economic, political and social) that are better measured at the group or environmental level (e.g., in relation to the availability of prenatal care) [8-18]. These mechanisms may also vary by other contextual factors. In particular, lower SEP can mean different things for families living in urban and rural settings [19]; for example, in Nova Scotia there is poorer access to specialized health services in rural setting [20]. Family composition may also change the implications of lower SEP since female-headed lone parent families are often young [21] and have low income [22,23], so it is likely harder to manage stressors than comparable two-parent families [24-29]. Identifying what dimensions of SEP mean at different levels and in different contexts can facilitate interventions [30]; yet, few studies have compared the consistency in associations across measures of SEP for perinatal outcomes, and across various contexts.

Most investigations into income disparities and perinatal outcomes have assumed that the effects of income on health are direct. As a result, these analyses often control for risk factors as potential confounders that may actually lie on the causal pathways that relate income level to adverse perinatal outcomes. By controlling for a partly or fully mediating factor, this approach leads to underestimates of the magnitude of income-related disparities. For example, mothers of lower SEP are more likely to smoke tobacco products than higher SEP mothers in Nova Scotia [31], and some findings suggest that lower SEP mothers may also be more likely to continue smoking during pregnancy due to the the stressfulness of their context for several reasons (e.g., , partly as a maladaptive coping habit [32]. Similarly, property values in Nova Scotia drive lower SEP families in Nova Scotia to live in more polluted environments in [33,34], so the deprivation level of a neighbourhood could reflect how likely individual are to be exposed to unhealthy environmental conditions [33,34]. In these examples, family and neighbourhood SEP may increase risk for adverse perinatal outcomes through pathways involving differential exposure to tobacco smoke and air pollution; thus, we argue that such risk factors should correctly be considered mediators of health effects related to income, rather than confounders.

This analysis asks: 1) “On which disease processes, in which subpopulations, and at what geographic levels can socioeconomic inequalities in perinatal health be demonstrated in Nova Scotia?”; and 2) “Do different indicators of SEP demonstrate varying magnitudes of inequalities?” We examined the relationship between several indicators of SEP at the household and neighborhood levels and adverse perinatal outcomes among singleton births in Nova Scotia between 1988 and 2003 using a population-based observational study of the SEP in the year of delivery and birth outcomes up to one year of life. Lastly, we examined whether income disparities varied across families in urban and rural settings, and in female-headed lone-parent families versus two-parent families. This analysis treats family income-related variables as proxies for SEP and assumes that other maternal risk factors for perinatal health may plausibly lie downstream of income on common causal pathways that shape the development and constraint of maternal health behaviours and exposures to environmental toxins.

## Methods

### Study population

The study population included all families (unit of analysis) that gave birth in Nova Scotia between 1988 and 2003. Data describing perinatal outcomes and maternal or household characteristics other than income were obtained from the Nova Scotia Atlee Perinatal Database (NSAPD), a registry that collects and compiles detailed maternal and perinatal health data for all births in the province by using trained personnel to extract information from antenatal and medical charts (as described elsewhere; [1]) in a reliable manner [35].

All singleton births in the province between 1988 and 2003 (n = 134 560) were included. Exclusions were then made where data were missing for key study variables, including income or any of the pregnancy outcomes (n = 16 632). Due to the low prevalence of several outcomes, enumeration areas with fewer than 50 births (n = 194) were excluded to avoid unstable or biased/unreliable estimates. The number of unique births included in this analysis was 117 734.

### Dependent variables

Adverse outcomes examined as dependent variables in this analysis include small- and large-for-gestational-age live birth (below the 3rd and greater or equal to the 90th percentile, respectively) [36], iatrogenic (i.e., delivery was induced or by cesarean delivery before the onset of labour) and spontaneous preterm birth (i.e., less than 37 weeks in both cases), perinatal death (including deaths between 22 weeks gestation and the end of the 7th day after delivery) and post-neonatal death (including deaths occurring from 28 through 364 days after birth).

### Independent variables

A partnership with Statistics Canada facilitated a confidential linkage with income tax-related information for the year in which delivery occurred using the T1 Family File [37]. The T1 Family File includes parent(s) and children living at the same address, but not persons living at the same address who are not in the family, including approximately 95% of all Canadians. The File aggregates income-related information from a variety of data recieved by the Canada Revenue Agency, including from all individuals who filed a tax return or who received a Canada Child Tax Benefit, children who filed a tax return and who reported the same address as their parent(s), as well as children and spouses who did not file their own tax return, but whose wage and salary information are available from other sources.

Specific independent variables examined as predictors of adverse perinatal outcomes included total family income, before and after tax (adjusted for family size and inflation, expressed in 2003 Canadian dollars; as described in Joseph et al. 2008 [1]); proportion of income from government transfers (including welfare payments, social security, and subsidies for businesses) as an indicator of relative reliance on redistributed income; total family income (after tax) below the Low Income Measure (LIM) as an indicator of poverty [38]; whether any income was derived from investments as an indicator of wealth [39]; and whether any contributions were made to a Registered Retirement Savings Plan (RRSP) as an indicator of middle social class [40].

An index of neighborhood deprivation describing the enumeration area of residence of families was calculated based on information from the 1986 Census using a previously described method [41]; this was the only independent variable included at the group level. The Atlee database was used to assign postal code of residence at the time of childbirth, which was linked to enumeration areas using the Statistics Canada Postal Code Conversion File. The deprivation index (range 0 to 5.3 across 606 enumeration areas, mean 1.8, SD 0.9) was converted into a percentile where higher values indicated higher levels of deprivation.

Other maternal and household characteristics that were reported in the NSAPD were examined as confounders: urban or rural place of residence and birth year; effect modifiers: urban or rural place of residence and single marital status (a proxy for female-headed lone parent family); or considered to be mediators and thus, not included in models measuring the full magnitude of income-related inequalities, including parity, pre-pregnancy weight, weight gain during pregnancy, maternal age, maternal smoking at delivery (a proxy for maternal smoking during pregnancy), gestational diabetes and prenatal class attendance.

### Data analysis

Multiple logistic regression was used to examine relationships between indicators of SEP and perinatal outcomes. We adjusted regression models for year of birth and residence in a rural or urban setting as potential confounders. Since our objective was to describe the magnitude of income disparities, we did not adjust for characteristics that may plausibly lie on the causal pathway between SEP and adverse perinatal outcomes (i.e., potential mediators), including parity, family parental composition, pre-pregnancy weight, weight gain during pregnancy, maternal age, maternal smoking at delivery, gestational diabetes, and prenatal class attendance [15].

In the second stage of the analysis, models for total family income (after tax) were further adjusted for neighborhood deprivation using fixed effects multilevel logistic regression models to examine the relative effects of household SEP and neighbourhood deprivation.

Modification of gradients for family income and neighborhood deprivation on perinatal outcomes by urban or rural place of residence, and by parental composition of families was assessed using a likelihood ratio test comparing a base model to a model where effects for family income and neighborhood deprivation were stratified by one or the other of these potential effect modifiers (α = 0.05).

All analyses were performed using SAS software (Cary, NC). The study was approved by the IWK Health Centre Research Ethics Board.

## Results

### Study population

Table 1 describes demographic characteristics and prevalence of adverse perinatal outcomes. Less than half of families (43.5%) resided in a rural postal code. A female lone-parent headed approximately 20% of families. In 43% of cases, the mother was nulliparous, while 20% had had at least two previous children. Seventy-three percent of mothers reported not smoking during pregnancy, while 21% reported smoking at least 10 cigarettes per day during pregnancy. Approximately 40% of mothers reported attending prenatal classes during pregnancy, which includes multiparous women who may have attended classes during earlier pregnancies.

**Table 1:** Maternal and family characteristics, income and adverse perinatal outcomes of 117734 singleton births in Nova Scotia, Canada, 1988-2003

		**Frequency**^**1 **^**(%)**
**Maternal characteristics**		
Residence in a rural postal code		
	Yes	51193 (43.48)
	No	66541 (56.52)
Birth year		
	1988-1990	29045 (24.67)
	1991-1993	23787 (20.2)
	1994-1996	25395 (21.57)
	1997-1999	18473 (15.69)
	2000-2003	21034 (17.87)
Parity		
	0	51002 (43.32)
	1	42775 (36.33)
	2	16813 (14.28)
	≥3	7144 (6.07)
Parental composition of family		
	Female-headed lone-parent family^2^	24642 (20.93)
	Two-parent family	93092 (79.07)
Pre-pregnancy weight (kg)		
	<55	25246 (23.99)
	55 - <75	58119 (55.22)
	75 - <90	14099 (13.40)
	≥90	7778 (7.39)
Weight gained during pregnancy (kg)		
	0 - ≤10.4	20497 (21.3)
	10.4 - ≤17.7	50348 (52.25)
	17.7 - ≤30	24444 (25.37)
	>30	1080 (1.12)
Age (years)		
	<20	8963 (7.61)
	20 - <25	26757 (22.73)
	25 - <30	40027 (34)
	30 - <35	30263 (25.7)
	35 - <40	10305 (8.75)
	≥40	1419 (1.21)
Smoking status (# of cigarettes per day at time of admission)		
	Non-smoker	79664 (73.07)
	1 - <10	8704 (8.0)
	10- 75	23029 (21.12)
	Known smoker, amount unknown	1651 (1.51)
Gestational diabetes		
	Yes	3156 (2.68)
	No	114578 (97.32)
Prenatal class attendance		
	Yes	40376 (39.69)
	No	61361 (60.31)
**Family income**		
Total family income (after tax)^3^		
	<$5,990.92	11773 (10.00)
	≥$5,990.92 < $9,119.4	11773 (10.00)
	≥$9,119.4 < $14,997.34	23547 (20.00)
	≥$14,997.34 < $20,759.95	23548 (20.00)
	≥$20,759.95 < $28,266.83	23546 (20.00)
	≥28,266.83	23547 (20.00)
Total family income (before tax)^3^		
	<$6,389	12125 (10.3)
	≥$6,389 - < $9,418	12203 (10.36)
	≥$9,418 - < $17,184	24176 (20.53)
	≥$17,184 - < $25,361	24031 (20.41)
	≥$25,361 - < $35,983	23313 (19.8)
	≥$35,983	21886 (18.59)
Total family income (after tax) below the low income measure^4^		10973 (9.32)
No income from investments		86310 (73.31)
No contribution to Registered Retirement Savings Plan		82735 (70.27)
**Adverse perinatal outcomes**		
Small-for-gestational-age live birth (<3rd percentile)		3934 (3.41)
Large-for-gestational-age live birth (>90th percentile)		15157 (13.13)
Iatrogenic pre-term birth (before 37 weeks)^5^		2316 (2.07)
Spontaneous pre-term birth (before 37 weeks)		4152 (3.66)
Post-neonatal death		197 (0.17)
Perinatal death		909 (0.77)

^1^Subject number varied due to missing values.

^2^Mother is not married or in a common-law relationship.

^3^Income adjusted for family size and inflation, expressed in 2003 Canadian dollars

^4^The Low Income Measure is fixed at 50% of median family income (after tax) adjusted for family size.

^5^Delivery was induced or by cesarean delivery before the onset of labour.

Total family income (after tax) was less than $20 760 in approximately 60% of families. Approximately 20% had an after tax family income at or above $28 267, and slightly fewer than 10% had after tax family income below the LIM. Fewer than a third of families received income from investments (27%) or made contribution to a RRSP (30%) during the year of delivery.

Large-for-gestational-age live birth was a relatively common outcome (13%), while perinatal and post-neonatal death were rare (prevalence of 0.8% and 0.2%, respectively). Small-for-gestational-age live birth occurred in 3.4% of cases, while iatrogenic and spontaneous preterm birth occurred in 2.1% and 3.7% of cases respectively.

### Magnitude of family income effects on adverse perinatal outcomes

**Figure 1:**
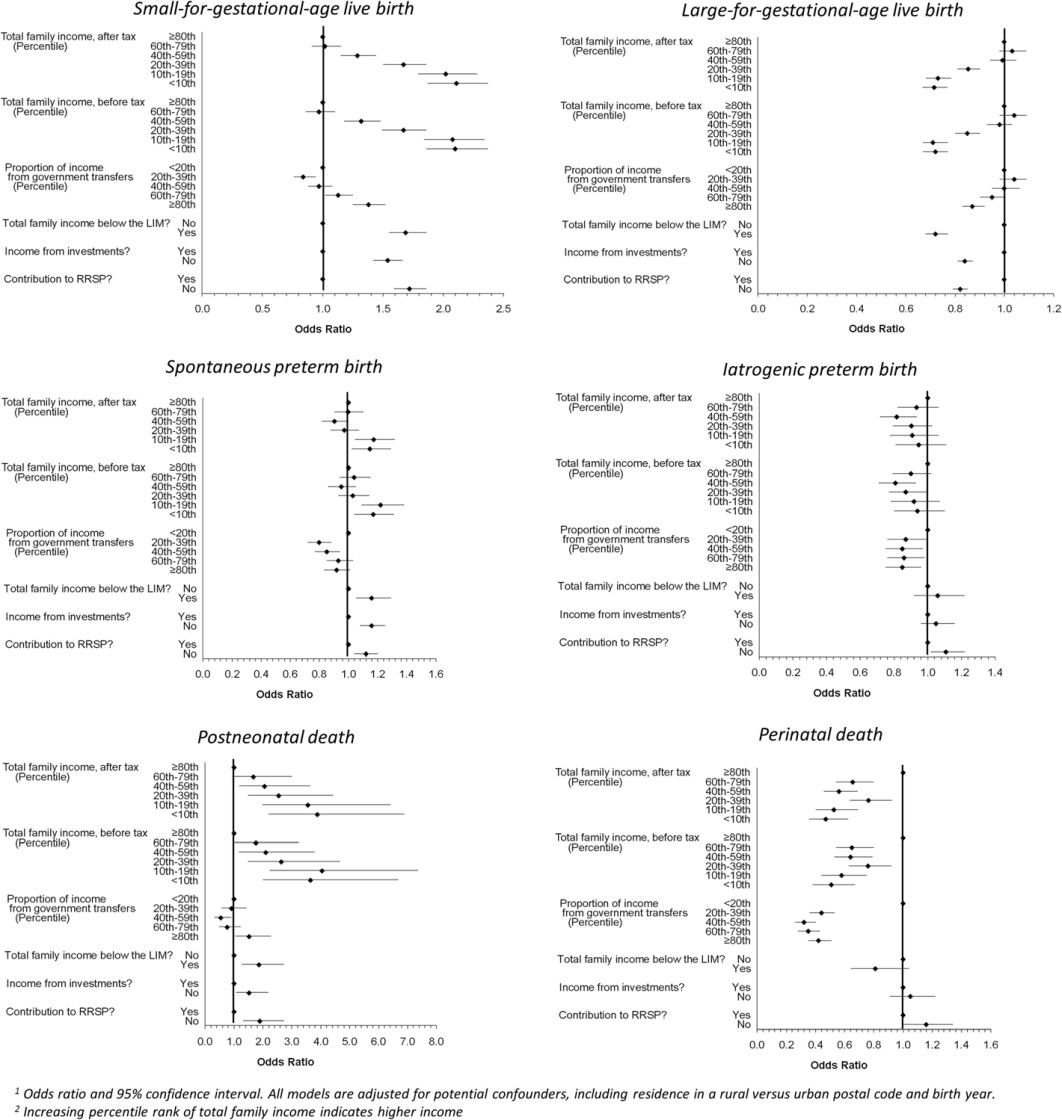
Relationship of family income characteristics with adverse perinatal outcomes among singleton births Nova Scotia 1988–2003.

Figure 1 presents the relationship between multiple indicators of SEP and adverse perinatal outcomes. There is a consistent association between lower SEP and higher odds ratios for SGA across all indicators of SEP, including a stepwise relationship for family income levels (before and after tax). For LGA, there was a similarly consistent pattern across all SEP indicators but in the opposite direction: that is, lower SEP was generally associated with protective odds ratios.

There was a consistent finding of higher odds ratios for postneonatal death across most indicators of low SEP, including a stepwise relationship with family income (before and after tax), with one exception. A U-shaped gradient with proportion of income from government transfers, was observed i.e., families with middle income (i.e., in the 40th to 50th percentile) had protective odds ratios for postneonatal death. Similarly, higher odds ratios for spontaneous preterm birth were found across most indicators of low SEP, although the relationship with family income was not stepwise, i.e., only families with income <20th percentile (before and after tax) had elevated odds ratios. Also, a U-shaped gradient for proportion of income from government transfers was observed, i.e., families with middle income (i.e., in the 20th to 59th percentile) had protective odds ratios for spontaneous preterm birth.

In contrast, there were inconsistent associations between indicators of SEP and iatrogenic preterm birth. A U-shaped gradient for total family income (before and after tax) was observed, i.e., families with income in the 20th to 79th percentile were associated with lower risk of iatrogenic preterm birth. There was a similar protective association for this outcome among all families with proportion of income from government transfers above the 20th percentile. Lack of investment income and RRSP contributions were not associated with this outcome.

Perinatal death showed significantly decreased risk for all lower income groups compared to the highest income group and for all groups of proportionate income from government transfers compared to the lowest proportion. Investment income, contributions to RRSP and total family income below the LIM demonstrated no associations with perinatal death.

### Effects of neighborhood deprivation on adverse perinatal outcomes

In models co-adjusted for total family income (after tax) (Table 2), higher levels of material deprivation in the neighborhood of residence were associated with increased risk for SGA, iatrogenic and spontaneous preterm birth and perinatal death. A U-shaped gradient appeared for LGA across quartiles of neighborhood deprivation, with relatively lower risk for families in the inter-quartile range. There was no association between neighborhood material deprivation and post-neonatal death.

**Table 2:** Relationship of total family income (after tax) and neighbourhood deprivation with adverse perinatal outcomes among 117734 singleton births in Nova Scotia, Canada, 1988-2003

**Perinatal outcomes**	**Socioeconomic domains**		**Crude OR (95% CI)**^**1**^	**Adjusted OR (95% CI)**^**1**^
Small-for-gestational-age live birth	Total family income (Percentile)			
		<10th (Lowest)	2.11 (1.87 - 2.37)	2.00 (1.78 - 2.26)
		10th-19th	2.02 (1.79 - 2.28)	1.91 (1.69 - 2.17)
		20th-39th	1.67 (1.50 - 1.86)	1.59 (1.42 – 1.77)
		40th-59th	1.29 (1.5 - 1.44)	1.25 (1.11 - 1.39)
		60th-79th	1.02 (0.91 - 1.15)	1.0 (0.89 - 1.12)
		≥80th (Highest)	1.00^2^	1.00^2^
	Neighbourhood deprivation index^3^ (Percentile)			
		≥75th (Lowest)	-	1.18 (1.07-1.30)
		50th-74th	-	1.23 (1.11-1.35)
		25th-49th	-	1.11 (1.01-1.23)
		<25th (Highest)	-	1.00^2^
Large-for-gestational-age live birth	Total family income (Percentile)			
		<10th (Lowest)	0.72 (0.67 - 0.77)	0.72 (0.67 - 0.78)
		10th-19th	0.73 (0.68 - 0.78)	0.74 (0.69 - 0.79)
		20th-39th	0.86 (0.82 - 0.91)	0.86 (0.82 - 0.91)
		40th-59th	0.99 (0.94 - 1.05)	1.0 (0.95 - 1.06)
		60th-79th	1.03 (0.98 - 1.09)	1.04 (0.98 - 1.06)
		≥80th (Highest)	1.00^2^	1.00^2^
	Neighbourhood deprivation index^3^ (Percentile)			
		≥75th (Lowest)	-	0.97 (0.92-1.02)
		50th-74th	-	0.92 (0.87-0.96)
		25th-49th	-	0.91 (0.87-0.95)
		<25th (Highest)	-	1.00^2^
Iatrogenic preterm birth	Total family income (Percentile)			
		<10th (Lowest)	0.95 (0.81 - 1.11)	0.91 (0.78 - 1.07)
		10th-19th	0.91 (0.78 - 1.06)	0.87 (0.74 – 1.02)
		20th-39th	0.90 (0.79 – 1.03)	0.87 (0.76 – 1.00)
		40th-59th	0.82 (0.72 - 0.93)	0.80 (0.70 - 0.91)
		60th-79th	0.94 (0.82 – 1.06)	0.92 (0.81 – 1.05)
		≥80th (Highest)	1.00^2^	1.00^2^
	Neighbourhood deprivation index^3^ (Percentile)			
		≥75th (Lowest)	-	1.15 (1.02-1.31)
		50th-74th	-	1.10 (0.97-1.24)
		25th-49th	-	1.05 (0.93-1.19)
		<25th (Highest)	-	1.00^2^
Spontaneous preterm birth	Total family income (Percentile)			
		<10th (Lowest)	1.15 (1.02 - 1.29)	1.09 (0.97 - 1.23)
		10th-19th	1.17 (1.05 - 1.32)	1.11 (0.99 - 1.25)
		20th-39th	0.97 (0.88 - 1.07)	0.93 (0.84 - 1.03)
		40th-59th	0.9 (0.82 – 1.0)	0.88 (0.79 - 0.97)
		60th-79th	1.0 (0.9 - 1.1)	0.98 (0.89 - 1.08)
		≥80th (Highest)	1.00^2^	1.00^2^
	Neighbourhood deprivation index^3^ (Percentile)			
		≥75th (Lowest)	-	1.16 (1.06-1.27)
		50th-74th	-	1.16 (1.06-1.28)
		25th-49th	-	1.01 (0.92-1.11)
		<25th (Highest)	-	1.00^2^
Perinatal death	Total family income (Percentile)			
		<10th (Lowest)	0.47 (0.36 - 0.62)	0.42 (0.31 - 0.55)
		10th-19th	0.52 (0.40 - 0.69)	0.46 (0.35 - 0.61)
		20th-39th	0.76 (0.63 - 0.92)	0.69 (0.56 - 0.83)
		40th-59th	0.56 (0.46 - 0.69)	0.52 (0.42 - 0.64)
		60th-79th	0.66 (0.54 - 0.80)	0.63 (0.52 - 0.76)
		≥80th (Highest)	1.00^2^	1.00^2^
	Neighbourhood deprivation index^3^ (Percentile)			
		≥75th (Lowest)	-	1.52 (1.25-1.85)
		50th-74th	-	1.32 (1.08-1.60)
		25th-49th	-	1.10 (0.91-1.34)
		<25th (Highest)	-	1.00^2^
Post-neonatal death	Total family income (Percentile)			
		<10th (Lowest)	3.89 (2.20 – 6.88)	3.82 (2.13 - 6.84)
		10th-19th	3.56 (1.98 – 6.41)	3.51 (1.93 – 6.39)
		20th-39th	2.55 (1.47 – 4.42)	2.51 (1.44 - 4.40)
		40th-59th	2.06 (1.18 – 3.63)	2.04 (1.16 - 3.60)
		60th-79th	1.67 (0.93 - 2.99)	1.66 (0.93 - 2.98)
		≥80th (Highest)	1.00^2^	1.00^2^
	Neighbourhood deprivation index^3^ (Percentile)			
		≥75th (Lowest)	-	1.10 (0.72-1.68)
		50th-74th	-	0.98 (0.63-1.53)
		25th-49th	-	1.14 (0.74-1.74)
		<25th (Highest)	-	1.00^2^

^1^Odds ratio and 95% confidence interval; models are adjusted for potential confounders, including residence in a rural versus urban postal code and birth year.

^2^Reference group.

^3^Neighbourhood deprivation index is comprised of data from the 1991 Census describing enumeration areas by: % 15+ without high school graduation,% lone-parent families,% homes needing major repairs,% 15+ unemployed,% families receiving government transfer payments, and % living below the low income cut-off (LICO).

In general, the pattern of effects by family income did not substantively change after controlling for neighborhood deprivation. The borderline significant increased risk for spontaneous preterm birth in the lowest quintile of family income was diminished and no longer statistically significant in co-adjusted models.

### Modification of effects by parental composition of family

**Figure 2:**
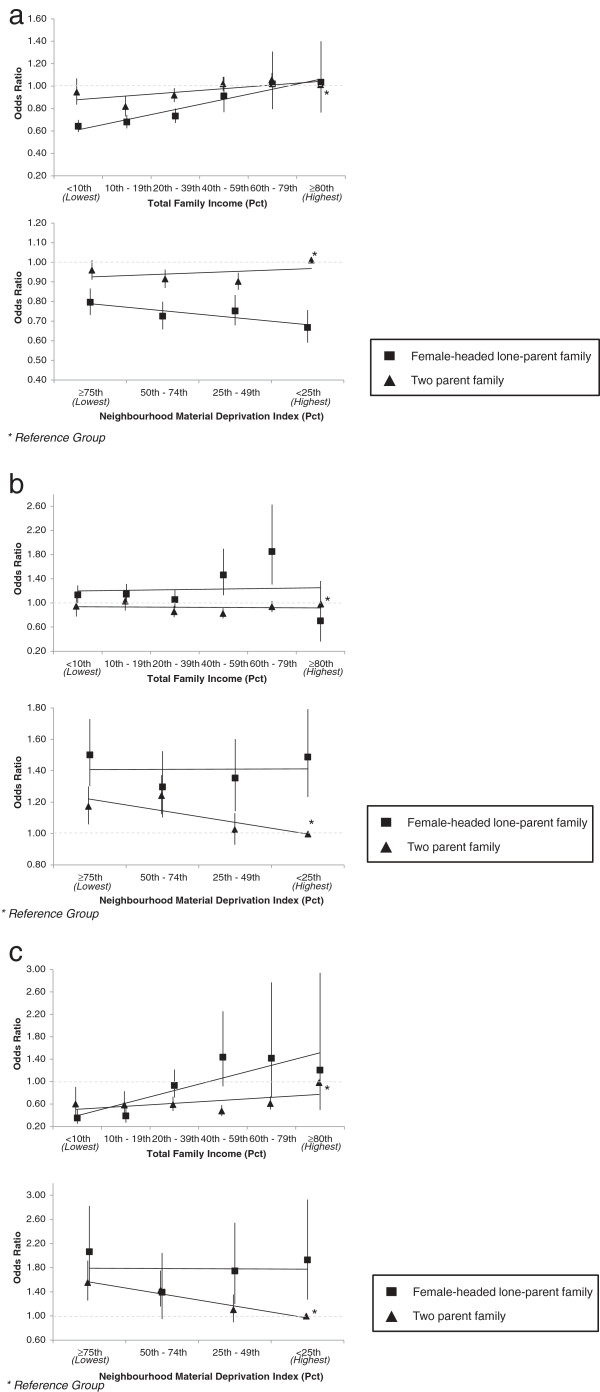
Perinatal disparities by parental composition of family members among singleton births, Nova Scotia 1988-2003 a. Large-for-gestational-age birth disparities; b. Spontaneous preterm birth disparities; c. Perinatal death disparities.

Figures 2a through Figure 2c present those outcomes for which there was a statistically significant improvement in model fit when stratifying effects in the co-adjusted model by parental composition of family. All patterns are visualized with two parent families having a total after tax income above the 80th percentile and living in neighbourhoods below the 25th percentile of neighbourhood material deprivation as the reference groups.

The protective gradient in LGA across lower total family income remained for families headed by a lone parent (Figure 2a), while no clear family income gradient in odds ratio was observed among two parent families. By contrast, a weak U-shaped pattern in odds ratios for material deprivation was found for LGA among two parent families, while a gradient of protective odds ratios emerged among female-headed lone parent families in neighbourhoods with lower deprivation.

In Figure 2b, odds ratios for spontaneous preterm birth among female-headed lone parent families were particularly elevated among those with total family income between the 40th and 79th percentiles. In contrast, Odds ratios for two parent families were under 1.0 and all 95% confidence intervals included 1.0 except for families at the 40-59th percentile. For neighborhood material deprivation, female-headed lone-parent families had uniformly high odds ratios across all levels of neighborhood deprivation. Odds ratios were elevated for two parent families as well but odds ratios decreased as neighborhood deprivation levels decreased.

Parental composition modified the effect of total family income on perinatal death (Figure 2c). Odds ratios for female-headed lone-parent families were protective for this outcome at the lowest end of total family income. For two parent families, odds ratio were uniformly below 1.0 for all income groups relative to two parent families above the 80th percentile. Modification of the effect of neighborhood material deprivation on perinatal death showed a very different pattern; odds ratios were uniformly high across all deprivation levels for female-headed lone-parent families, whereas among two parent families, odds ratios were only elevated for those residing in neighborhoods with high levels of deprivation.

### Modification of effects by place of residence

**Figure 3:**
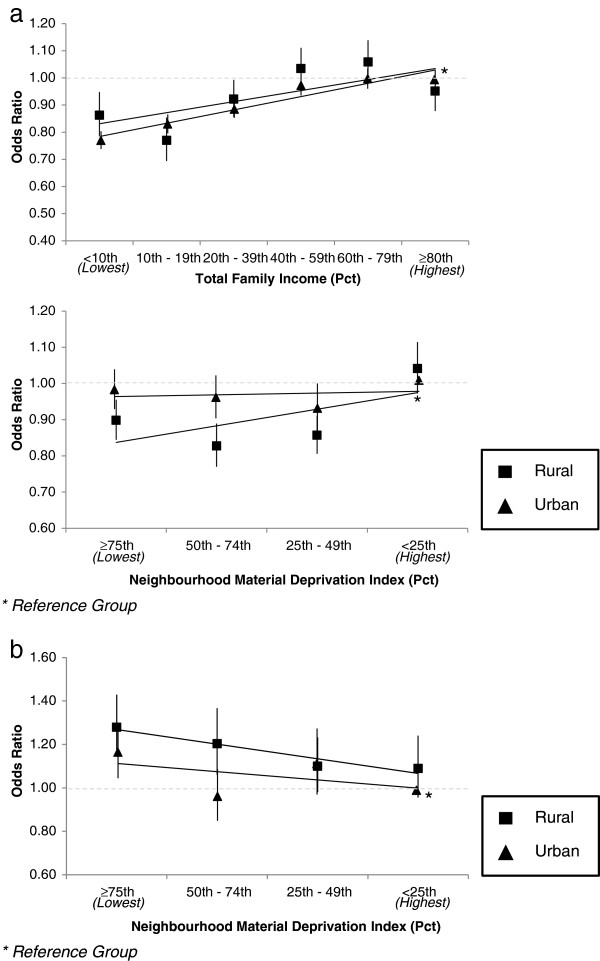
Perinatal disparities by place of residence among singleton births, Nova Scotia 1988-2003 a. Large-for-gestational-age birth disparities; b. Spontaneous preterm birth disparities.

Figures 3a and b highlight statistically significant effect modification in household and neighbourhood gradients by urban and rural place of residence, with urban families having a total after tax income above the 80th percentile and living in neighbourhoods below the 25th percentile of neighbourhood material deprivation as the reference groups.

While a stepwise decrease in odds ratios for large-for-gestational-age live birth was observed across decreasing levels of family income in the total population, Figure 3a indicates a steeper positive income gradient among rural compared to urban families. Odds ratios for large-for-gestational-age live birth were larger in urban neighborhoods with higher deprivation, while there were generally protective odds ratios for living in higher deprivation neighborhoods in urban settings.

Figure 3b indicates increased odds ratios for spontaneous preterm birth in neighbourhoods of higher deprivation. Odds ratios were significantly elevated for urban families with deprivation above the median; where odds ratios were only significantly elevated for rural families in the highest quartile of neighbourhood deprivation.

## Discussion and conclusions

We sought to explore the relationship between SEP, measured using different indicators at the family and neighborhood levels, and perinatal outcomes in this population-based sample that was linked to a rich set of indicators on income and census data. Our findings suggest that the income-related indicators did not always have consistent patterns of association with the perinatal outcomes, and in some instances, such as for spontaneous preterm birth, had contradictory findings. Both individual level income and neighborhood level deprivation, when considered together, were significant predictors for most of the perinatal outcomes we examined.

For all outcomes examined in this study, there were consistent, nearly identical patterns for total family income after tax and before tax with all perinatal outcomes. The other individual-level SEP variables did not always have relationships to the outcomes that mirrored that of the total family income variables. For example, having a medium proportion of income from government transfers was risk-protective for spontaneous preterm birth and for postneonatal death; whereas lower income based on other variables conferred higher risk for these outcomes. While most income variables likely reflect the resources available to the families, a variable like proportion of income from government transfers did not take into account the absolute family income levels which may, in part, be contributing to the different patterns for SGA, spontaneous preterm and postneonatal death.

While the addition of neighborhood deprivation to the regression model did not substantively change the gradient for total family income (after tax) for any outcomes, the patterns of association were not always consistent across the individual and neighborhood level variables. For example, risk for perinatal death and iatrogenic preterm birth was substantially lower for those with lower total family income compared to those in the highest income category, while higher levels of neighborhood deprivation were associated with elevated risk for these outcomes. Such findings suggest that the two indicators of SEP may be operating through different pathways, at least for those outcomes. Neighborhood deprivation likely measured a wide range of social variables that went beyond income levels of neighborhood residents (e.g., see [30,42]). Further research is needed to delineate the relevant pathways and mechanisms involved in these discordant findings.

Our findings also illustrated how family composition and level of urbanicity modify the associations between SEP and the some of the outcomes. The patterns of association between SEP and LGA, spontaneous preterm birth and perinatal death varied depending on family composition or urbanicity. This too suggests that the importance of certain pathways and mechanisms may vary by context.

These findings are from a Canadian setting with single payer universal health coverage for all essential healthcare services, so we expect there to be some generalizability for other settings with universal coverage like the United Kingdom. Whereas in other settings like the United States, where the quality of antenatal care may be commensurate with the level of family income, we might expect income to be a stronger determinant of perinatal outcomes, and so we might expect inequalities to be of a larger magnitude than we report.

There were limitations of our data and approach. First, not all birth records were linked to data on the income variables; linkage was successful in 81.3% of records [43]. Thus, the extent to which the failure to link may have contributed to an over or underestimate of the associations is not known. Also, some families were missing data on SGA (2%), LGA (2%) and preterm birth outcomes (iatrogenic, 5%; spontaneous 4%). Families excluded from the analysis here were less likely to be of higher SEP or to reside in lower deprivation neighbourhoods. For all outcomes, the difference in income and deprivation composition within strata was less than 7%, and usually less than 4% (data not shown). It is not possible to predict the direction of bias; however, given the small size of these differences, any impact on the magnitudes reported here are likely to be minimal.

Second, the use of cross sectional data meant that data on total family income (after tax) were for the same year as the year of birth, raising questions about the direction of the association; however, for studies of pregnancy it is unlikely that the outcome influenced the levels of income for the family. Finally, while SEP may reflect a range of causal mechanisms at the group level in health research, including the level of material deprivation, income inequality, social capital and racial segregation (e.g., [42]), we only assessed the magnitude of inequalities relative to a measure of material deprivation in the neighbourhood in this study.

Our main conclusions are that the choice of income indicator may influence the magnitude and pattern of inequality observed. In some instances, such as for iatrogenic and spontaneous preterm birth, some of the indicators of individual level SEP may contradict each other in terms of whether higher income is risk inducing or protective for the outcome. Thus, choosing indicators of SEP that are consistent with the purpose of the investigation is critical for the examination of inequities in perinatal health. Also, because the patterns of inequities may vary by context--family composition or urbanicity--examination of effect modification should be a priority in future studies. As with all analyses that demonstrate gaps and inequalities, a critical next step is to investigate why these inequalities exist and what factors, with intervention potential, can be identified so that effective programs and policies can be designed and implemented. This is but a first step in that larger research agenda to uncover strategies to address to continue the trend of reducing the gap in in perinatal health in Canada [44].

## Abbreviations

LGA: Large for gestational age; LIM: Low income measure; NSAPD: Nova scotia atlee perinatal database; RRSP: Registered retirement savings plan; SEP: Socioeconomic position; SGA: Small for gestational age.

## Competing interests

The authors declare that they have no competing interests.

## Authors’ contributions

KS conceptualized and designed the analyses, oversaw the interpretation of findings, conducted the statistical analysis, drafted the initial manuscript, and reviewed and revised the final manuscript. POC conceptualized and designed the analyses, assisted with interpretation of findings, and reviewed and revised the final manuscript. JM conducted the background literature review, revised and edited the manuscript and finalized the submission for publication. LD gave input into the design of the analyses, assisted with interpretation of findings, and reviewed and revised the final manuscript. JF gave input into the design of the analyses, assisted with interpretation of findings, and reviewed and revised the final manuscript. KSJ gave input into the design of the analyses, assisted with interpretation of findings, and reviewed and revised the final manuscript. VMA gave input into the design of the analyses, assisted with interpretation of findings, and reviewed and revised the final manuscript. All authors read and approved the final manuscript.

## Pre-publication history

The pre-publication history for this paper can be accessed here:

http://www.biomedcentral.com/1471-2393/14/96/prepub
